# Functional Characterization of *PeMep* Gene Reveals Its Roles in the Vegetative Growth, Stress Adaptation, and Virulence of *Penicillium expansum*

**DOI:** 10.3390/foods14111908

**Published:** 2025-05-28

**Authors:** Juanying Huang, Chenyang Zhu, Mengyue Wu, Guanghao Li, Luning Zhao, Xiaoshuang Xia, Yun Wang

**Affiliations:** 1 School of Food and Biological Engineering, Jiangsu University, Zhenjiang 212013, China; 2222218061@stmail.ujs.edu.cn (J.H.); 2232218005@stmail.ujs.edu.cn (C.Z.); 2222318079@stmail.ujs.edu.cn (M.W.); 2222318075@stmail.ujs.edu.cn (G.L.); 1000005022@ujs.edu.cn (L.Z.); 2 Center of Analysis, Jiangsu University, Zhenjiang 212013, China; 1000005374@ujs.edu.cn

**Keywords:** *Penicillium expansum*, effector, metalloprotease, stress tolerance, pathogenicity

## Abstract

*Penicillium expansum*, a major postharvest pathogen, causes blue mold decay in apples, resulting in substantial economic losses and mycotoxin contamination. Despite the importance of effector proteins in fungal pathogenicity, the role of metalloproteases in *P. expansum* remains unclear. Here, we characterize an effector candidate, PeMep, through whole genome sequencing and functional analyses. Functional validation confirmed the secretory capacity of its signal peptide via yeast assays and subcellular localization. Deletion of *PeMep* significantly impaired fungal growth (23% reduction), conidiation (23.3% decrease), and germination efficiency. The Δ*PeMep* mutant exhibited hypersensitivity to osmotic, oxidative, and thermal stresses, highlighting its vital role in environmental adaptability. Importantly, pathogenicity assays revealed attenuated virulence in the Δ*PeMep* mutant, with 15–30% smaller lesion sizes on apples and delayed hyphal penetration compared to the wild-type, demonstrating that PeMep is essential for the pathogenic process of *P. expansum* 3.3703. These findings identify PeMep as a potential multifunctional effector protein crucial for fungal development, environmental adaptation, and pathogenicity in *P. expansum* 3.3703, providing a novel target for postharvest disease management.

## 1. Introduction

*Penicillium expansum* (*P. expansum*), a devastating postharvest pathogen of pome fruits, causes blue mold disease in apples and pears, leading to substantial economic losses and food safety risks globally [1]. Liu et al. employed scanning electron microscopy (SEM) to characterize the infection mechanism, demonstrating that *P. expansum* conidia directly penetrate host tissues through germ tube-mediated invasion and degrade apple cell walls [2]. Further transmission electron microscopy (TEM) observations revealed that *P. expansum* initially disrupts the outer cell walls of apple fruits, then invades the inner cell walls, damaging intracellular structures [2,3]. This progressive destruction eventually leads to tissue maceration and decay. Beyond tissue maceration, this fungus produces patulin—a mycotoxin classified as a Group 3 carcinogen by the International Agency for Research on Cancer [4] —which accumulates in infected fruits and processed products, posing severe threats to human health [5]. Therefore, further exploration of virulence factors from *P. expansum* and the molecular mechanisms underlying its pathogenicity is critical for developing novel strategies to control this fungal disease.

During plant infection, pathogens secrete effector molecules to facilitate colonization. Effectors play pivotal roles in host/pathogen interactions, and their ability to manipulate host defenses determines the success of pathogen colonization [6]. Recent studies have focused on identifying effectors contributing to the virulence of *P. expansum* pathogenicity and decay progression in apple fruits [3,7]. *P. expansum* secretes over 80 proteases [8], which serve two primary roles: facilitating nutrient acquisition to support fungal growth and counteracting protein-based defense responses in apple fruits [9,10]. Levin et al. [11,12] demonstrated that knocking out the subtilisin-related peptidase genes *Peprt* and *Penlp1* in *P. expansum* impaired conidiation, altered hyphal morphology, and reduced lesion diameters on apple fruits. With the availability of whole-genome sequences for *P. expansum* and other fungal species [8,13], secretomic approaches—a subfield of proteomics—have been widely employed to study fungal secretory proteins, shedding light on potential mechanisms of pathogen/host interactions [14,15]. For instance, Yao et al. [16] analyzed the genome and transcriptome of *Heterodera schachtii*, bioinformatically predicting 51 candidate effector proteins. Functional validation of 37 candidates revealed 13 effectors suppressing Bax-induced cell death and 9 effectors inhibiting both Bax- and GPA2/RBP1-triggered cell death [16]. Similarly, Liu et al. conducted a comprehensive genome-wide screening of *Magnaporthe oryzae*, predicting 577 candidate-secreted effectors, of which 3 were experimentally confirmed to exhibit significant roles in pathogenicity [17]. Effector proteins are typically characterized by small molecular size, presence of signal peptides, absence of transmembrane domains, and high cysteine content. Leveraging these traits for bioinformatic prediction, combined with integrated analyses of genomic, transcriptomic, and proteomic data, enables the identification of numerous potential effectors [18,19].

To date, a number of effectors belonging to the metalloprotease family have been identified in phytopathogenic fungi. Among these, zinc metalloproteases are pivotal in mediating pathogen/host interactions. For example, the zinc metalloprotease ProA in *Legionella pneumophila* suppresses host Toll-like receptor 5 (TLR5) signaling, dampens inflammatory responses, and, thereby, evades immune defenses to promote bacterial proliferation [20]. Similarly, FgFly1, a zinc metalloprotease in *Fusarium graminearum*, interacts with the host TaCAMTA protein to positively regulate fungal virulence [21]. Most members of the M35 family are zinc-dependent metalloproteases secreted by pathogens and serve as key effectors. For instance, *Magnaporthe oryzae*’s M35 effector Avr-Pita interacts with the rice Pi-ta protein to trigger host immunity [22]. *Fusarium oxysporum* employs FoMep1 to cleave chitin-binding domains of tomato chitinases [23], neutralizing antifungal activity, while *Rhizoctonia cerealis* RcMEP2 induces host oxidative bursts and suppresses defense gene expression in wheat [24]. Similarly, entomopathogenic fungi like *Metarhizium robertsii* utilize M35 proteases to disrupt insect immune responses [25]. Despite growing recognition of metalloprotease functions in other pathogens, their roles in *Penicillium* species remain underexplored.

In this study, we combined whole-genome data and canonical effector features to screen for candidate genes potentially involved in *P. expansum* 3.3703′s pathogenicity. We identified and characterized *PeMep*, a secretory M35 metallopeptidase in *P. expansum* 3.3703. The *PeMep* knockout mutant exhibited impaired growth, attenuated virulence on apple fruits, and compromised stress tolerance. This work fills a critical gap in understanding metalloprotease-mediated virulence mechanisms in *P. expansum*, provides new insights into the molecular basis of postharvest fruit diseases, and establishes a foundation for elucidating the role of *PeMep* in pathogenesis.

## 2. Materials and Methods

### 2.1. Fungal Strains and Culture Conditions

The fungal strain employed in this study, *Penicillium expansum* strain 3.3703 (*P. expansum* 3.3703), was obtained from the China General Microbiological Culture Collection Center (CGMCC, Beijing, China) and maintained on potato dextrose agar (PDA) plates in the dark at 25 °C for 5–7 days.

### 2.2. Whole-Genome Sequencing and Effector Candidate Genes Screening

Genomic DNA was extracted from 7-day-old cultures using the SanPrep Column Plasmid Mini-Preps Kit (Sangon Biotech Co., Ltd., Shanghai, China) and quantified with a Nanodrop One (Thermo Fisher Scientific Inc., Waltham, MA, USA). The library preparation and next-generation sequencing were furnished by Sangon Biotech Co., Ltd. (Shanghai, China). First, 500 ng of quantified DNA was randomly fragmented by Covaris (Woburn, MA, USA). Next, the Hieff NGS^®^MaxUp II DNA Library Prep Kit for Illumina^®^ (Yeasen Biotechnology Co., Ltd., Shanghai, China) was used for the next steps. Briefly, Endprep enzyme was added to the repair end and 3′ end A tail ligation. Then, the adaptor was ligated by an enhancer and Fast T4 DNA (Sangon Biotech Co., Ltd., Shanghai, China) ligase. An index primer was added by PCR and the amplified product, about 400 bp, was selected by DNA selection beads. Library quality was verified using a Qubit (v4.0) fluorometer before Illumina^TM^ platform sequencing [26]. Raw data were quality-checked with FastQC (v0.11.2), filtered, and assembled de novo using Falcon and CANU [27], with error correction by Pilon (v1.18). Assembly completeness was validated via BUSCO (v3.0.2) [28].

Gene prediction was performed using GeneMark-ES (v4.35) and GlimmerHMM (v3.0.4), while repetitive sequences were identified with RepeatModeler (v1.0.4) and annotated via RepeatMasker (v4.0.5). Functional annotation involved BLAST+ (v2.2.28) alignment against CDD (https://ftp.ncbi.nlm.nih.gov/pub/mmdb/cdd/cdd.tar.gz, accessed on 28 December 2023), KOG (ftp://ftp.ncbi.nih.gov/pub/cog/kog/, accessed on 28 December 2023), COG (http://www.ncbi.nlm.nih.gov/COG/, accessed on 28 December 2023), NR (https://ftp.ncbi.nlm.nih.gov/, accessed on 28 December 2023), NT (https://ftp.ncbi.nlm.nih.gov/, accessed on 28 December 2023), Pfam (http://pfam.xfam.org/, accessed on 28 December 2023), Swiss-Prot (http://web.expasy.org/docs/swiss-prot_guideline.html, accessed on 28 December 2023), and TrEMBL (https://www.uniprot.org/, accessed on 28 December 2023) databases [29]. GO terms were assigned based on Swiss-Prot/TrEMBL homology, and KEGG (Kyoto Encyclopedia of Genes and Genomes, http://www.kegg.jp, accessed on 28 December 2023) pathways were annotated using KAAS (v2.1) [30]. For phylogenetic analysis, predicted ITS sequences were aligned against the NCBI nt database (identity >95%), with the top 30 matches used to construct a maximum likelihood tree in FastTree (v2.1.11).

In silico candidate effector genes were predicted according to the previously described method [31,32]. In brief, *P. expansum* 3.3703 secreted protein genes were predicted using the SignalP program (https://services.healthtech.dtu.dk/service.php/SignalP-6.0, accessed on 28 December 2023) to determine the presence of the N-terminal signal peptide. Subsequently, TargetP (https://services.healthtech.dtu.dk/service.php/TargetP-2.0, accessed on 28 December 2023) and TMHMM (https://dtu.biolib.com/Deep/TMHMM, accessed on 28 December 2023) programs were used to identify and remove proteins predicted to be located at the mitochondrion and membrane. Finally, proteins containing fewer than four cysteine residues were excluded from the candidate effector pool.

Total RNA was extracted from *P. expansum* 3.3703-inoculated apple tissues using the RC411-C1 Plant RNA Extraction Kit (Sangon Biotech Co., Ltd., Shanghai, China). RNA was quantified, reverse-transcribed into cDNA using HiScript II QRT SuperMix (Vazyme Biotech Co., Ltd., Nanjing, China), and subjected to qRT-PCR on a StepOne Plus system (Applied Biosystems, Foster City, CA, USA) with SYBR Green Master Mix (Sangon Biotech Co., Ltd., Shanghai, China). The target transcripts included the *PeMep* gene and the internal control gene 18S rDNA. Amplification was performed under the following conditions: denaturation at 95 °C for 30 sec, 40 cycles of 95 °C for 10 sec, 60 °C for 30 sec, and a melt curve step. Primer pairs for the target genes were designed using the Integrated DNA Technologies (IDT, v5.3.1) online tool (https://www.idtdna.com/, accessed on 26 January 2024), with sequences provided in Supplementary Table S1. Three biological and technical replicates were included.

### 2.3. Construction of the Phylogenetic Tree for PeMep Protein

The amino acid sequence of the PeMep protein was aligned with sequences from the NCBI database, and the top 15 protein sequences with the highest similarity were selected. Sequence alignment was performed using MEGA 11 software, and a phylogenetic tree was constructed using the neighbor-joining method.

### 2.4. Yeast Signal Sequence Trap Assay

The yeast signal peptide screening was used to verify the secretory function of effector protein signal peptides [33]. The yeast strain chosen for the experiment was YTK12 [34]; it lacks the sucrose convertase gene and cannot grow on a medium with sucrose as the only carbon source. The predicted signal peptide was inserted into the vector pSUC2. Transfer of recombinant plasmid to yeast strain YTK12(Genotype: Δ*suc2*, *trp1*, Coolaber Technology Co., Ltd., Beijing, China) screened positive clones in CMD-W medium (deletion of tryptophan, Coolaber Technology Co., Ltd., Beijing, China). Positive clones grown on CMD-W medium were transferred to a YPRAA machine (2% raffinose as the sole carbon source, Coolaber Technology Co., Ltd., Beijing, China) and incubated upside down at 28 °C. The function of the signal peptide was screened and verified by a YPRAA medium and a TTC (2,3,5Triphenyltetrazolium Chloride, Coolaber Technology Co., Ltd., Beijing, China) coloration test. Further, the signal peptide function was validated by chromogenic reaction.

### 2.5. Subcellular Localization Analysis

To generate the recombinant plasmid 35s:PeMep, the *P. expansum* 3.3703 gene was amplified using the primers P1300-PeMep-SacI-R and P1300-PeMep-SaII-F to obtain the *PeMep* gene fragment flanked by SacI and SalI restriction sites. The fragments were cloned into the pCAMBIA1300-35S-EGFP vector (HonorGene Biotech, Changsha, China). The recombinant plasmids were then transformed into *Agrobacterium tumefaciens* GV3101. These strains were used to infect 4-week-old *Nicotiana benthamiana* leaves by Agrobacterium-mediated transformation, as described previously [35]. Following Agrobacterium infiltration, plants were kept in darkness for 12 h. Fluorescence signals in the infiltrated regions of *N. benthamiana* leaves were examined at 40 h after inoculation (hai) using a Olympus FV1000 confocal laser scanning microscope (Olympus Corporation, Tokyo, Japan) [36]. Green fluorescence from enhanced GFP (eGFP) was detected using a 488 nm confocal microscope.

### 2.6. Generation of PeMep Deletion Mutant

*PeMep* deletion mutant (Δ*PeMep*) was generated by homologous recombination according to Zhang et al. [32]. Approximately 600–700 bp upstream and downstream fragments of the *PeMep* gene were amplified from the *P. expansum* 3.3703 genomic DNA using PeMep-UF/PeMep-UR and PeMep-DF/PeMep-DR primer pairs, respectively. Hygromycin B resistant gene (hyg) fragment gene was amplified from pCAMBIA-1300 plasmid using hyg-F/hyg-R primer pair. The three fragments were joined to generate the gene deletion cassette by overlap extension PCR using the primer pair PeMep-knock-F/PeMep-knock-R. The amplified products were transformed into *P. expansum* 3.3703 protoplast using a polyethylene glycol (PEG)-mediated transformation. Potential positive transformants were selected through resistance screening using a PDA medium containing hygromycin (120 μg/mL). The obtained hygromycin-resistant colonies were further confirmed by PCR using the specific primer pairs PeMep-out-F/R and hyg-F/R, which were designed to detect the hygromycin resistance cassette that replaced the PeMep gene in the deletion mutant, and PeMep-in-F/R primer pair, which was specific to the *PeMep* gene. The nucleotide sequences of *PeMep*, upstream, and downstream regions, along with the amino acid sequence of PeMep, are provided in Table S2. Primer pairs for this study were designed with the IDT online tool and SnapGene v6.2.2 software, synthesized by Sangon Biotech (Shanghai, China), and their sequences are listed in Table S1.

### 2.7. Fungi Morphology and Growth Assays

Wild-type and Δ*PeMep* strains were inoculated in PAD plates and cultured at 25 °C for 7 days. Conidial suspensions from each strain were then adjusted to a concentration of 10^7^ conidia/mL using sterile distilled water, with concentration validation through hemocytometer counts.

To assess radial growth, 30 μL aliquot of conidial suspensions from wild-type and Δ*PeMep* strains were centrally inoculated on PDA plates. The plates were incubated at 25 °C under dark conditions, with colony diameters measured daily for 7 days after inoculation (dai).

Sterilized coverslips were inserted at a 45° angle into PDA plates (3 mm thickness). A 3 μL aliquot of conidial suspension was inoculated at the coverslip/agar interface. Hyphal morphology was observed under a Nikon Eclipse E200 microscope (Nikon Corporation, Tokyo, Japan) at 24, 48 and 72 hai.

A 100 μL aliquot of conidial suspension was inoculated onto PDA plates and incubated at 25 °C in darkness for 12 h. Germination rates were determined microscopically by enumerating germinated conidia. A conidium was considered germinated when the germ tube length exceeded the spore diameter.

### 2.8. Stress Assay

In order to detect the sensitivity of wild-type and Δ*PeMep* trains to cell wall inhibitors, oxidative stress, and osmotic stress, both strains were cultured in PDA medium separately supplemented with 0.7 g/L Congo Red, 0.4 g/L SDS, 1.2 M NaCl, 1.2 M KCl, 1.5 M Sorbitol, 0.015 M H_2_O_2_,0.003 M ZnSO_4_, or 0.3 M MgSO_4_ [37]. The diameter was measured after 5 days of incubation at 25 °C.

To evaluate thermal stability, conidial suspensions (10^7^ conidia/mL) were subjected to heat treatment at 45 °C and 4 °C for 1 h, respectively. Subsequently, 0.1 mL aliquots from each treatment group were uniformly spread on PDA plates (3 mm thickness). The inoculated plates were incubated at 25° C, and conidial germination was periodically assessed under optical microscopy at 6, 8, 10, and 12 hai.

### 2.9. Virulence Assay

Apple fruits were surface-sterilized with 3% (*w*/*v*) NaClO for 5 min, rinsed thrice with sterile distilled water, and air-dried. Uniform 5-mm-diameter wounds were created at the equatorial zone of each fruit using a sterile tool. Each wound was inoculated with 30 μL conidial suspension (10^7^ conidia/mL) of the targeted strains. Inoculated fruits were placed in plastic baskets sealed with transparent film to maintain 90% relative humidity and stored at 25 °C [38]. Disease severity was quantified by measuring lesion diameters throughout the storage period.

### 2.10. Electron Microscopy

The inoculation method for apple fruits was performed as described in the virulence assay protocol. Fruit discs (10 mm diameter × 5 mm thickness) were excised from wound margins at 0, 6, 8, 12, and 14 hai. The specimens were fixed with 2.5% (*v*/*v*) glutaraldehyde at 4 °C for 12 h. Subsequently, sequential dehydration was conducted using graded ethanol series (25%, 50%, 70%, 95%, and 100%), as described by Zhang et al. [32]. Dehydrated samples were mounted on aluminum stubs, sputter-coated with gold/palladium, and examined under an S-4800 field emission scanning electron microscope (JEOL Ltd., Tokyo, Japan).

### 2.11. Statistical Analysis

The quantitative data in this study were expressed as means *±* standard deviation (SD). The significance between the data was assessed using the Student’s *t*-test, and significance levels were defined as follows: * *p* < 0.05, ** *p* < 0.01, and *** *p* < 0.001.

## 3. Results and Discussion

### 3.1. Genome-Wide Analysis and Screening of Effector Candidate Genes

The genome assembly of *P. expansum* 3.3703 was 32,108,221 base pairs (bp) in size, containing 41 ambiguous bases and 136 contigs. The average contig length was 236,089.86 bp, with the largest contig spanning 2,208,060 bp. Assembly continuity was further evidenced by N50 and L50 values of 803,615 bp and 13, respectively. The GC content was calculated at 48%. These genomic characteristics align closely with previously reported values for *P. expansum* [8,39], which exhibit genome sizes ranging between 31.0 and 35 Mb and GC contents of approximately 47–48%. The high continuity and minimal presence of unresolved bases collectively validate the completeness and reliability of this genome assembly.

**Figure 1:**
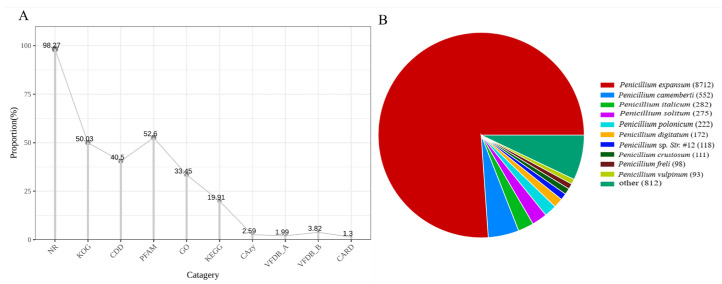
Genomic features of *P. expansum* 3.3703. (**A**) Proportion of genes annotated against major databases (NR, COG, KOG, Pfam, Swiss-Prot, TrEMBL). (**B**) Distribution of homologous species based on BLAST analysis. The sector area represents the quantity of sequences aligned to each species (larger areas correspond to higher sequence counts), with species names annotated in the legend.

Among the annotated genes, the NR database yielded the highest number of functionally classified genes (11,447 genes, accounting for 98.27% of the total predicted genes; Figure 1A, Table S3). Comparative genomic analysis revealed that 8127 sequences showed significant homology to *P. expansum* (74.8% of the total annotated genes; Figure 1B), indicating a high degree of conservation between *P. expansum* 3.3703 and the reference strain of *P. expansum*. Phylogenetic analysis based on ITS sequences further demonstrated that *P. expansum* 3.3703 exhibited close evolutionary relationships with *Penicillium expansum* (accession FJ491785), *P. italicum* (accession HQ850920), and other phylogenetically proximal strains (Figure S1).

**Figure 2:**
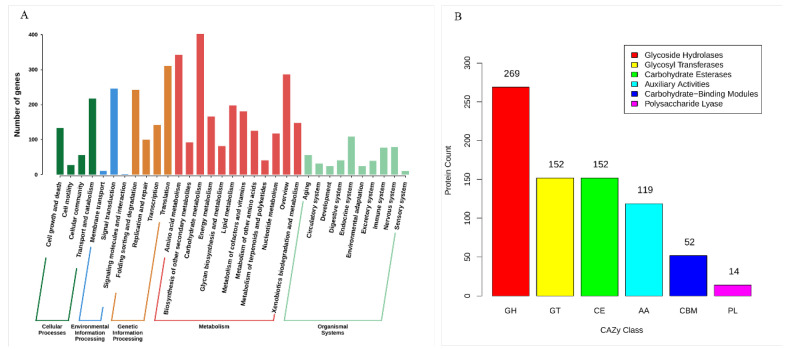
Functional annotation in *P. expansum* 3.3703. (**A**) KEGG pathway classification of annotated genes. (**B**) CAZyme family distribution, highlighting glycosyl hydrolases (GHs) as the dominant class.

Based on the KEGG database, this study linked genes to metabolic pathways through KO (KEGG Orthology) annotation and performed hierarchical functional classification. As shown in Figure 2A, the annotation results revealed that *P. expansum* 3.3703 harbors a high number of genes annotated under the “metabolism” functional module. Notably, carbohydrate metabolism and amino acid metabolism pathways contained the highest gene counts, which may be associated with its saprophytic lifestyle, enabling growth and nutrient acquisition on decaying fruit surfaces [10,40,41].

The CAZymes annotation of the *P. expansum* 3.3703 genome (Figure 2B) revealed a carbohydrate-active enzyme profile dominated by glycoside hydrolases (GHs) (269 members), followed by glycosyltransferases (GTs) and carbohydrate esterases (CEs) (152 each). Phytopathogenic fungi secrete GHs as effectors to degrade plant cell walls, facilitating nutrient acquisition and suppression of host immune signaling during colonization [42,43]. The GHs in *P. expansum* 3.3703 likely serve similar functions. For instance, Zhou et al. demonstrated that the Δ*Peclg* mutant (lacking a GH family glucanase-encoding gene) exhibited significantly reduced virulence and growth on apple fruits compared to the wild-type strain [44]. The enrichment of the GH family in *P. expansum* 3.3703 suggests that it may primarily rely on GHs to degrade plant cell wall polysaccharides, driving tissue maceration to promote pathogenesis.

During host infection, phytopathogenic fungi secrete diverse effector proteins to manipulate host immune defenses for successful colonization [3,45]. These effectors are typically secretory proteins, prompting us to prioritize genome-wide identification of secreted proteins. Following the previously described methodology, 116 candidate effector genes were identified through rigorous screening (Table S4). Notably, over half (66.37%) of these candidates encode hypothetical proteins of unknown function. Among the biochemically annotated proteins, enzymes dominated the effector pool including glycosyl hydrolases and peptidases—a pattern consistent with findings in *P. expansum* effectoromics studies [3,10,38].

Further qRT-PCR revealed that the candidate effector gene ctg00012_0005619_t (designated *PeMep*, *Penicillium expansum* Metalloprotease) was significantly upregulated in *P. expansum* 3.3703 during apple fruit infection at 6 hai and 9 hai (Figure S2), suggesting that *PeMep* is likely involved in the host infection process. Therefore, we selected this candidate effector for further functional characterization.

### 3.2. Characterization of the Effector Candidate PeMep in P. expansum 3.3703

**Figure 3:**
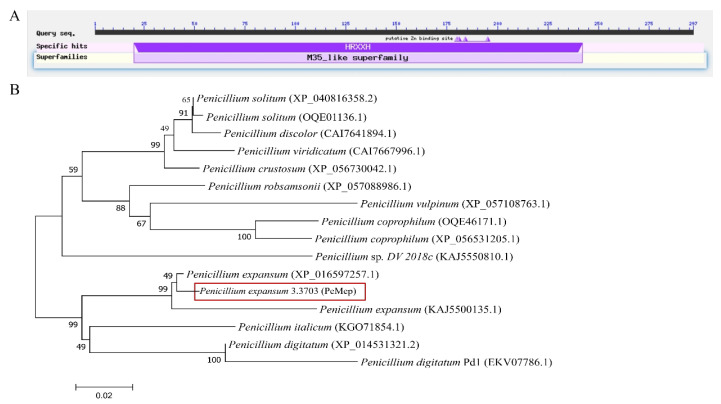
Bioinformatic analysis of PeMep. (**A**) Functional domain analysis of PeMep, showing the conserved M35 metallopeptidase motif (HRXXH). (**B**) Phylogenetic tree of PeMep.

Bioinformatic analysis of the NCBI database identified the effector candidate gene *PeMep* encoding a zinc-dependent M35 metalloprotease with a conserved catalytic HRXXH motif (Figure 3A). Using NCBI, we identified *P. expansum* sequences with high sequence similarity (>80%) to the PeMep protein of strain 3.3703. A phylogenetic tree constructed with MEGA 11 (Figure 3B) revealed that the PeMep protein from strain 3.3703 shares high homology with another *P. expansum* M35 protease (XP_016597257.1) and has 98% sequence identity (Bootstrap = 99).

**Figure 4:**
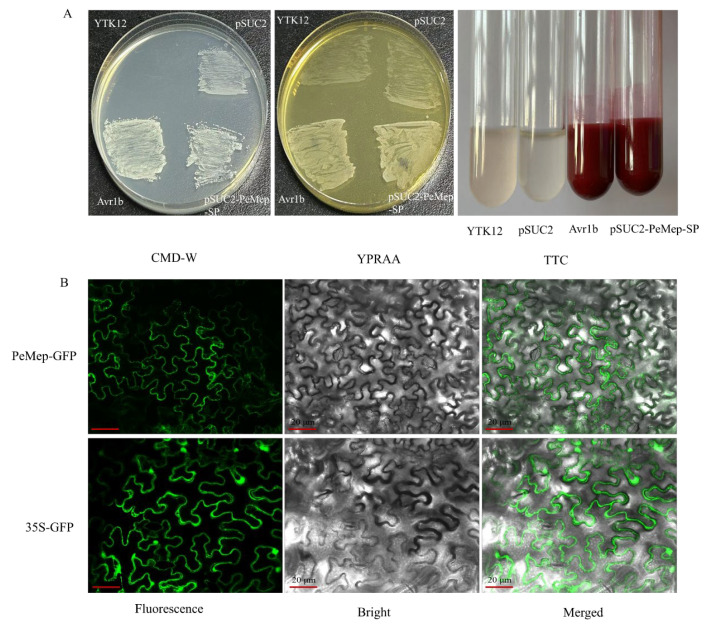
Characterization of PeMep. (**A**) Validation of PeMep signal peptide functionality using the yeast YTK12 strain. Growth on YPRAA medium and TTC chromogenic reaction confirm secretory activity. CMD-W: Tryptophan-deficient medium; YPRAA: Raffinose medium; TTC: Color reaction solution; YTK12: Plasmid-free control strain; pSUC2: Empty vector negative control; Avr1b: Positive control (**B**) Subcellular localization of PeMep-GFP fusion protein in *N. benthamiana* epidermal cells. Scale bar: 20 μm.

According to the molecular mechanism of the classical secretory pathway, effector proteins typically possess a functional N-terminal signal peptide as a core characteristic, which serves as the essential molecular signature initiating the secretory process [46]. SignalP 6.0 predicted a 15 amino-acid secretory signal peptide (MLLLNLILSAAIAAA) at the N-terminus of PeMep. To further validate the secretory activity of the signal peptide, a yeast secretion assay system was employed to assess its functionality. Functional validation using the yeast signal peptide trap system demonstrated that the pSUC2-PeMep-SP construct enabled normal growth of the YTK12 strain on the YPRAA medium, indicating that the PeMep signal peptide facilitated extracellular secretion of invertase for raffinose utilization [33]. Furthermore, the 2,3,5-triphenyltetrazolium chloride (TTC) chromogenic reaction further confirmed that the PeMep signal peptide directed invertase secretion (Figure 4A), corroborating its secretory functionality and suggesting that the PeMep protein is likely secreted extracellularly to exert its role. Subcellular localization analysis revealed that the PeMep candidate effector predominantly accumulates in the apoplastic space of host plant cells (Figure 4B). Collectively, these findings indicate that during pathogen infection, the PeMep protein is secreted into the extracellular space and suggest its potential targeting of the host apoplast to exert its function.

Members of the M35 metalloprotease family are widely distributed among pathogenic fungi, where they exhibit functional diversity as effectors during host infection. Most M35 family proteins are zinc-dependent metalloproteases secreted by pathogens. For instance, Avr-Pita, an M35 member from *Magnaporthe grisea*, acts as an effector that interacts with the Pi-ta protein in plant cells to trigger host defense responses [22]. Similarly, *Rhizoctonia cerealis* RcMEP2 and *Metarhizium anisopliae* M35-4 demonstrate secretory activity and function as virulence factors during host colonization [24,25]. In the case of *P. expansum*, the causal agent of blue mold in apples, PeMep—a homolog of the M35 family—likely plays a comparable role during infection.

Yeast signal sequence trapping and subcellular localization assays demonstrated that *PeMep* likely functions in the host extracellular space via the canonical secretory pathway. This finding is consistent with prior studies on M35-family effectors that found that *Verticillium dahliae* effectors VdM35-1 and VdASPF2 trigger immune responses by targeting plant plasma membrane components [47], while *Rhizoctonia cerealis* effector ReMEP2 is secreted into the apoplast of *N. benthamiana* leaves to induce cell death [24]. Notably, truncation of the secretory signal peptides in VdM35-1, VdASPF2, and ReMEP2 completely abolished their cell death-inducing activity, further underscoring the indispensable role of secretion machinery in effector functionality.

### 3.3. Role of PeMep in Fungal Growth and Development

**Figure 5:**
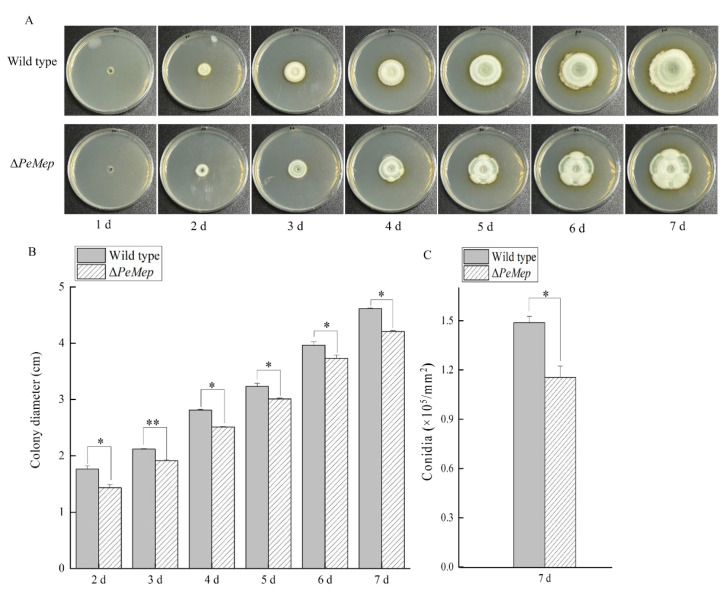
Phenotypic defects of Δ*PeMep* mutant. (**A**) Colony morphology of wild-type and Δ*PeMep* strains on PDA after 7 days at 25 °C. (**B**) Radial growth rates of wild-type and Δ*PeMep* measured daily (*n* = 3). (**C**) Conidial production quantified using a hemocytometer (*n* = 3). Data: mean ± SD; *t*-test: * *p* < 0.05, ** *p* < 0.01 vs. wild-type.

**Figure 6:**
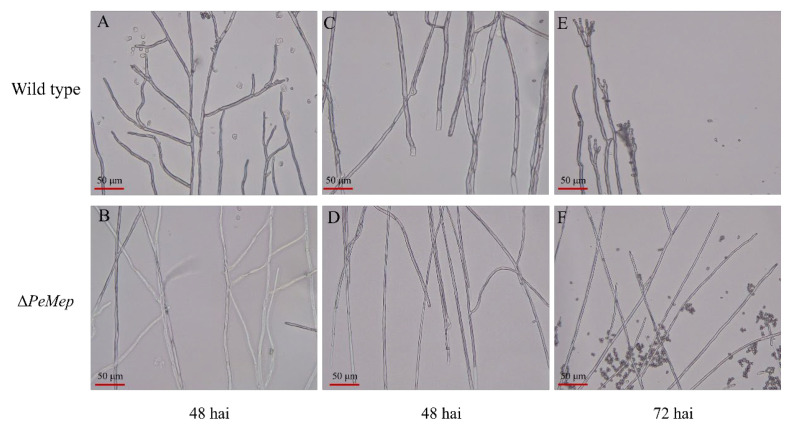
Hyphal morphology of wild-type and Δ*PeMep*. (**A**,**B**) Mid-region hyphae at 48 h after inoculation (hai). (**C**,**D**) Hyphal branching at basal regions (48 hai). (**E**,**F**) Hyphal tips at 72 hai. Δ*PeMep* exhibits reduced septation and aberrant branching. Scale bars: 50 μm.

**Figure 7:**
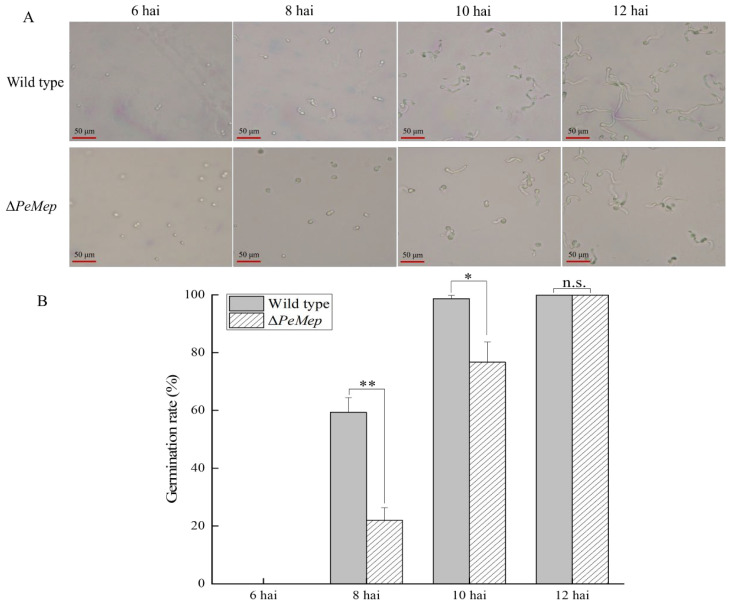
Germination dynamics of wild-type and Δ*PeMep* conidia. (**A**) Microscopic images of germinated conidia at 6, 8, 10, and 12 h after inoculation (hai). (**B**) Germination rates quantified at each time point (*n* = 3). Data: mean ± SD; *t*-test: * *p* < 0.05, ** *p* < 0.01 vs. wild-type; n.s.: not significant. Scale bars: 50 μm.

To investigate the role of *PeMep* in fundamental biological processes such as vegetative growth, morphogenesis, and spore germination in *P. expansum* 3.3703, we constructed the *PeMep* gene knockout mutant (Δ*PeMep*) via homologous recombination (Figure S3) and conducted phenotypic characterization of the mutant strain. Colony morphology analysis revealed that Δ*PeMep* formed compact, gear-like hyphal margins (Figure 5A), contrasting with the fluffy, radially expanding wild-type colonies. Quantitatively, Δ*PeMep* displayed a 23% reduction in growth rate and a 23.29% decrease in conidiation (Figure 5B,C). Microscopic observations further demonstrated impaired hyphal branching and a complete absence of conidiophore formation in the mutant (Figure 6). Germination assays highlighted delayed initiation and reduced efficiency in Δ*PeMep*; while wild-type conidia achieved full germination by 10 hai, the mutant achieved only 76.67% germination at the same time point (Figure 7).

The developmental defects in Δ*PeMep*—including stunted hyphal growth, delayed conidial germination, and reduced sporulation—highlight the critical role of the M35 metalloprotease PeMep in *P. expansum* 3.3703 morphogenesis. These phenotypes align with metalloprotease-related developmental disruptions observed across fungal species. For instance, deletion of the HRXXH-motif-containing VdASPF2 in *Verticillium dahliae* impairs conidial septation and maturation [47], while the zinc-dependent metalloprotease FpMep in *Fusarium proliferatum* positively regulates hyphal growth and conidiation [48], with its knockout reducing sporulation by 30.8%. Similarly, *Metarhizium robertsii* relies on M14-family metalloproteases Mrmep1/Mrmep2 for hyphal integrity and conidial separation [49]. Notably, plant-derived hevein-like antimicrobial peptides suppress *Fusarium* growth by targeting fungal metalloproteases, mirroring Δ*PeMep*’s hyphal defects [50]. Collectively, these underscore metalloproteases as key regulatory factors in fungal differentiation, with PeMep representing a critical M35-family regulator of growth and asexual reproduction in *P. expansum* 3.3703.

### 3.4. Functional Versatility of PeMep in Environmental Stress Adaptation

**Figure 8:**
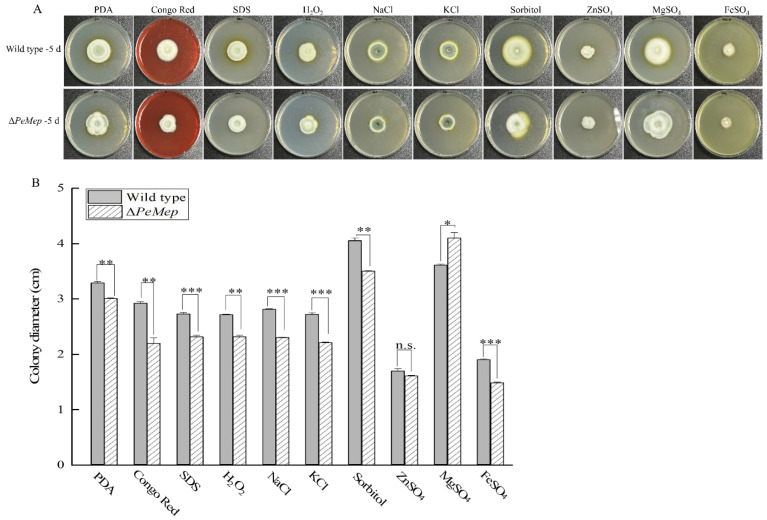
Stress sensitivity assays. (**A**) Colony growth of wild-type and Δ*PeMep* under osmotic (NaCl, KCl, sorbitol), oxidative (H_2_O_2_), cell wall-disrupting (Congo Red, SDS), MgSO_4_, ZnSO_4_, and FeSO_4_ stressors. (**B**) Colony diameters after 5 days (*n* = 3). Data: mean ± SD; *t*-test: * *p* < 0.05, ** *p* < 0.01, *** *p* < 0.001 vs. wild-type; n.s.: not significant.

Phytopathogenic fungi need to overcome various environmental stresses during host invasion. To investigate whether the *PeMep* gene is involved in environmental stress adaptation in *P. expansum* 3.3703, both the wild-type strain and Δ*PeMep* mutant strain were inoculated onto PDA medium supplemented with stress agents. Their growth was observed, and colony diameters were measured to assess phenotypic responses. The results showed that the Δ*PeMep* displayed hypersensitivity to diverse stressors (Figure 8). Under Congo Red (cell wall stress), colony diameters were reduced by 24.7% compared to wild-type, while SDS (membrane disruption) caused a 14.8% decrease. Oxidative stress (H_2_O^2^) and metal toxicity (FeSO_4_) inhibited growth by 14.4% and 21.9%, respectively. High salinity (NaCl, KCl) further suppressed growth by 17.8–18.7%, highlighting *PeMep*’s role in osmotic adaptation. Intriguingly, Δ*PeMep* displayed contrasting responses to sorbitol and MgSO_4_: under sorbitol stress, its growth was inhibited by 13.4%, whereas MgSO_4_ supplementation enhanced its growth by 13.6%, suggesting that *PeMep* may play a dual role in osmoregulation.

**Figure 9:**
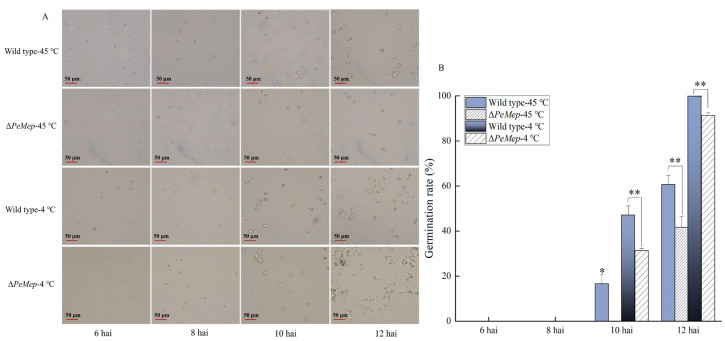
The effects of temperature on conidial germination of the wild-type and Δ*PeMep*. (**A**) Germination of conidia treated with high temperature (45 °C) and low temperature (4 °C) for 1 h, observed at 6–12 h after treatment. (**B**) Germination rates (*n* = 3). Data: mean ± SD; *t*-test: * *p* < 0.05, ** *p* < 0.01 vs. wild-type.

Thermal stress assays revealed Δ*PeMep*’s vulnerability to temperature extremes. After 45 °C treatment, Δ*PeMep* spores showed no germination at 10 hai versus 16.7% for wild-type and only 41.7% germination at 12 hai (vs. 60.7% for wild-type; Figure 9). Under 4 °C, Δ*PeMep* germination lagged at 10 hai (31.3% vs. 47.0%) and reached only 91.3% at 12 hai (vs. 100% for wild-type).

These findings suggest that beyond its direct role in pathogenesis (Section 3.5), *PeMep* likely exhibits functional versatility, contributing to environmental stress adaptation. Existing studies have shown that fungal effector proteins frequently exhibit functional pleiotropy, coordinating virulence through both host manipulation and stress resilience mechanisms. For instance, in *Fusarium oxysporum*, deletion of the M35 effector FocM35_1 not only reduces virulence but also impairs growth under oxidative (H_2_O_2_) and osmotic stress (high salinity), whereas the wild-type thrives under these conditions [51]. Similarly, *Verticillium dahliae* requires VdM35-1 and VdASPF2 to mitigate osmotic stress (NaCl, KCl, sorbitol) [47,52]; their deletion renders the fungus hypersensitive to SDS, Congo Red, and osmotic stress, thereby compromising pathogenicity.

We propose that M35 proteases may indirectly promote virulence by shielding pathogens from host-derived ROS or hyperosmotic environments. Our data corroborate this hypothesis: Δ*PeMep* showed exacerbated growth inhibition under H_2_O_2_ and high salinity compared to the wild-type, confirming *PeMep*’s role in conferring antioxidant capacity and osmotolerance (Figure 8). Furthermore, delayed spore germination under temperature extremes highlights *PeMep*’s contribution to thermotolerance (Figure 9). Such broad-spectrum stress adaptability implies that *PeMep* enhances fungal fitness not only during host infection but also in natural or postharvest environments, where it may counteract diverse abiotic stressors to ensure survival.

### 3.5. PeMep Is Essential for the Virulence of P. expansum 3.3703

**Figure 10:**
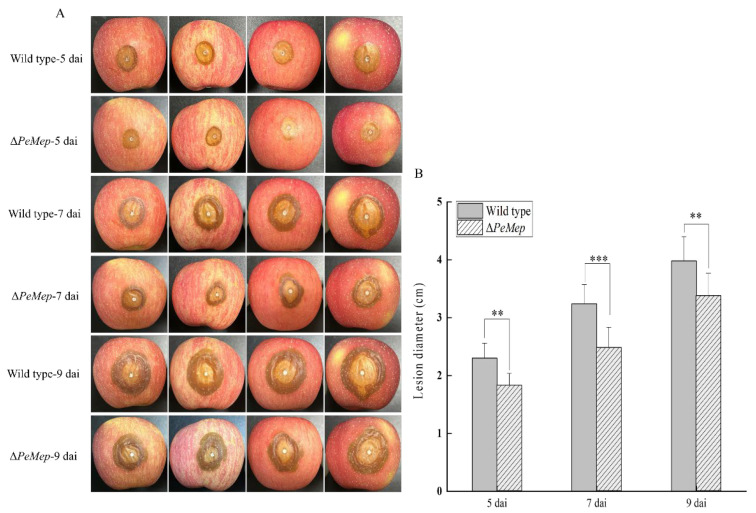
Virulence assay on apple fruits. (**A**) Lesion progression caused by wild-type and Δ*PeMep* at 5, 7, and 9 days after inoculation (dai). (**B**) Lesion diameters measured at each time point (*n* = 5 fruits). Data: mean ± SD; *t*-test: ** *p* < 0.01, *** *p* < 0.001 vs. wild-type.

**Figure 11:**
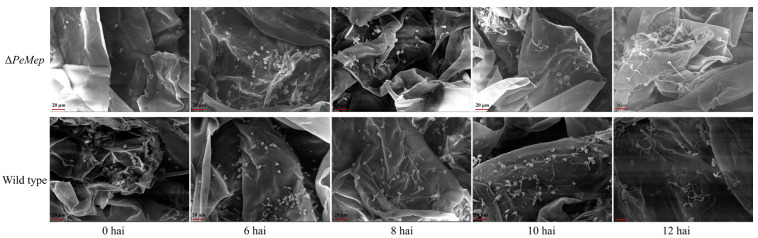
Scanning electron microscopy (SEM) of apple tissue infection. Δ*PeMep* shows delayed germination and impaired penetration. Scale bars: 20 μm.

Pathogenicity assays on apple fruits demonstrated Δ*PeMep*’s attenuated virulence, with lesion diameters 15–30% smaller than wild-type at 5–9 days after inoculation (Figure 10). SEM analysis of inoculated apple tissues further demonstrated that Δ*PeMep* exhibited delayed conidial germination at 6 hai, whereas wild-type conidia had initiated germination. By 8 hai, Δ*PeMep* hyphae showed impaired penetration ability compared to the wild-type (Figure 11).

Pathogenicity assays demonstrated that the *PeMep* gene knockout mutant exhibited significantly reduced virulence on apples, indicating that *PeMep* is a secreted protein closely associated with the pathogenic process of *P. expansum* 3.3703. The absence of this protein compromised the fungal infection capability. This phenotype aligns with observations in M35 effector mutants of other phytopathogenic fungi. For instance, the secreted M35 metalloprotease effector FocM35_1 in *Fusarium oxysporum* interacts with and inhibits banana chitinase activity [51], and its deletion attenuates virulence in banana wilt pathogens. Similarly, the M35 effector RcMEP2 in *Rhizoctonia cerealis* (wheat sharp eyespot pathogen) induces host cell death and suppresses chitinase gene expression, enhancing wheat susceptibility; deletion of *RcMEP2* diminishes pathogenicity [24]. In *Verticillium dahliae*, effectors VdM35-1 and VdASPF2 trigger reactive oxygen species (ROS) bursts and activate plant immunity, leading to cell death, and their deletion reduces virulence [47].

We hypothesize that the attenuated virulence of Δ*PeMep* may stem from its inability to counteract apple defense responses during early infection. Upon detecting fungal invasion, apples likely initiate defense responses. The absence of *PeMep* may impair the fungus’s capacity to neutralize these defenses, thereby restricting growth. SEM further revealed that *PeMep* knockout significantly inhibited *P. expansum* 3.3703 conidial germination and hyphal penetration within host tissues. Previous experiments demonstrated that *PeMep* regulates fungal growth, stress adaptation, and conidiation. Deletion of *PeMep* reduced conidial production, delayed germination, and compromised environmental resilience, collectively impairing host colonization. These deficiencies likely synergistically hinder hyphal penetration, weaken infectivity, and slow lesion expansion.

## 4. Conclusions

This study identified 116 candidate effectors in *P. expansum* 3.3703 through whole-genome sequencing and bioinformatics analysis, with a functional focus on *PeMep*, a candidate effector gene from the M35 metalloprotease family. Experimental validation confirmed that PeMep is a secreted protease, with its EGFP fusion protein localized to the apoplast of *N. benthamiana*, indicating its extracellular mode of action. The knockout strain Δ*PeMep* exhibited significantly reduced growth rate, conidial production, and germination efficiency, along with heightened sensitivity to osmotic, oxidative, and thermal stresses, demonstrating *PeMep*’s critical role in the development and environmental adaptation of *P. expansum* 3.3703. Virulence assays revealed that Δ*PeMep*-infected apples developed notably smaller lesions, while SEM further showed impaired spore penetration ability into host tissues. These findings establish that PeMep participates in vegetative growth, stress adaptation, and host infection processes in *P. expansum* 3.3703, serving as a potential multifunctional effector candidate. This work enriches the theoretical framework of *P. expansum* infection mechanisms and provides a scientific foundation for developing postharvest disease control strategies in apples.

## Data Availability

The data presented in this study are available on request from the corresponding author.

## Supplementary Materials

The following supporting information can be downloaded at: https://www.mdpi.com/article/10.3390/foods14111908/s1, Figure S1: Phylogenetic tree constructed from ITS sequences; Figure S2: qRT-PCR analysis of *PeMep* relative expression levels in apple fruit tissues at differ-ent time points after inoculation; Figure S3: Diagrammatic representation of Deletion of *PeMep* in *P. expansum* 3.3703; Table S1: Primers; Table S2: Information Related to *PeMep* Protein; Table S3: annotation_summary; Table S4: Predicted effector protein.
