# Supplementary material for: Functional Characterization of PeMep Gene Reveals Its Roles in the Vegetative Growth, Stress Adaptation, and Virulence of Penicillium expansum
Source: Foods. 2025 May 28;14(11):1908. doi: 10.3390/foods14111908 (PMC12154198; doi:10.3390/foods14111908)
Supplement: Supplementary file 1 [file foods-14-01908-s001.zip › figure S1~S3.pdf]

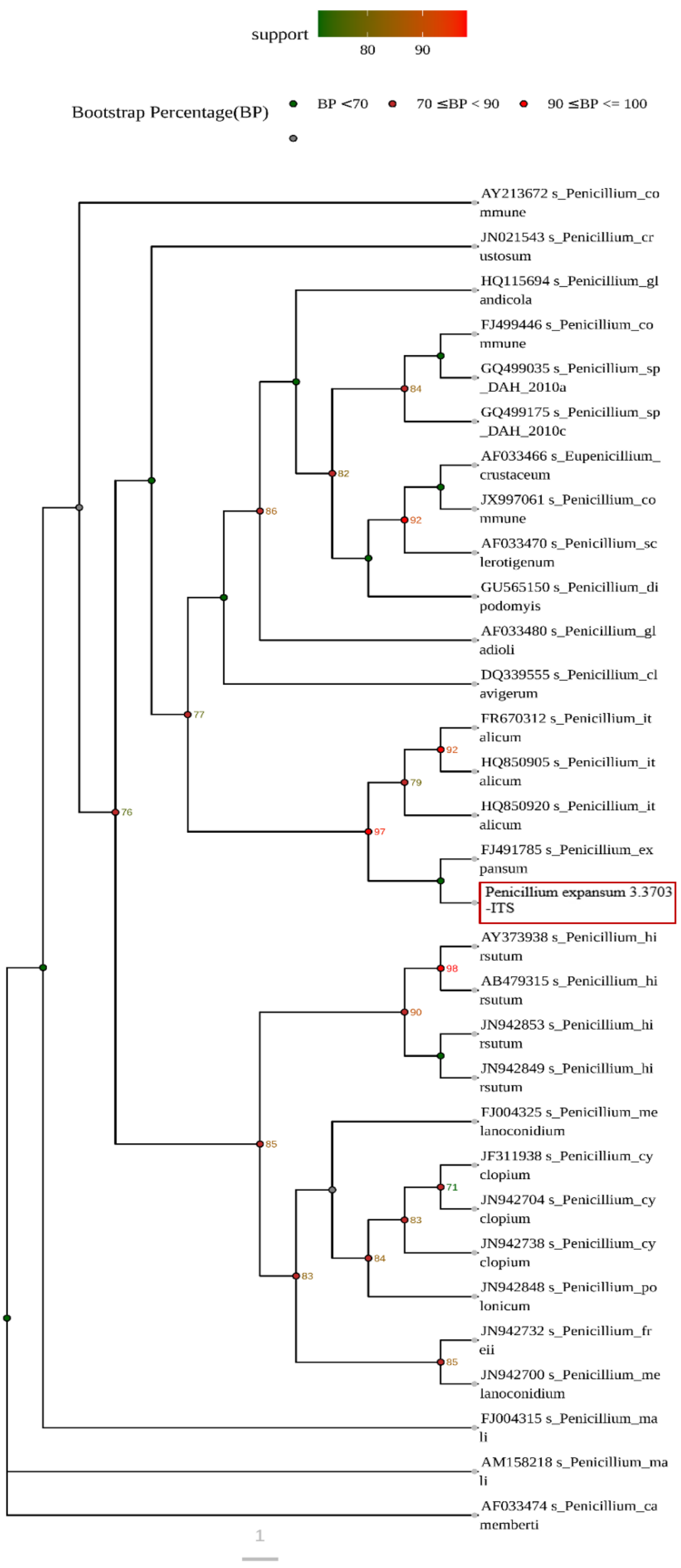

Figure S1. Phylogenetic tree constructed from ITS sequences, showing evolutionary relationships between *P. expansum* strain 3.3703 and related species. Bootstrap values (>70%) are indicated at nodes.

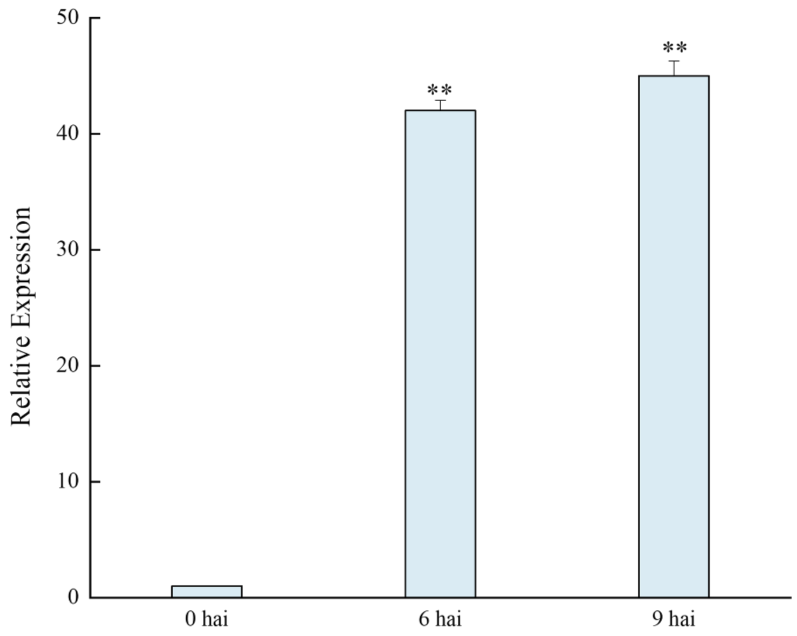

Figure S2. qRT-PCR analysis of *PeMep* relative expression levels in apple fruit tissues at different time points after inoculation (hai). Asterisks indicate significant differences compared to 0 hai (**$P < 0.01$). The 18S rDNA gene was used as an endogenous control. Data represent mean ± SD from three independent biological replicates.

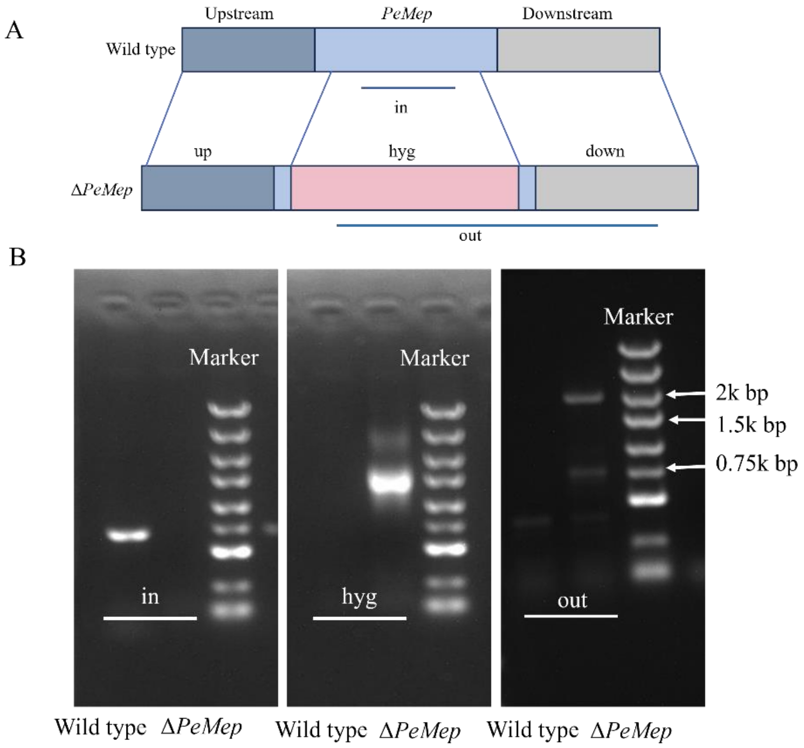

Figure S3. Diagrammatic representation of Deletion of *PeM35* in *P. expansum* 3.3703. (A) Schematic diagram illustrating the construction principle of the *PeMep* gene knockout mutant. The knockout strategy was based on homologous recombination, where left and right homologous arms were designed to replace the *PeMep* gene sequence. The fusion fragment carries the hygromycin resistance gene (*hyg*) as a selection marker. Upon transformation, the target gene was disrupted via a double-crossover recombination event. (B) Verification of Δ*PeMep* deletion strains with PCR. Primers hyg-F/R and PeMep-out-F/R amplified the expected bands exclusively in mutant strains, with no product detected in wild-type controls. The absence of amplification using PeMep-in-F/R primers in mutants demonstrates that the PeMep gene was precisely replaced by the hygromycin cassette without non-specific insertions.
