# Supplementary figures and images for: Functional Characterization of PeMep Gene Reveals Its Roles in the Vegetative Growth, Stress Adaptation, and Virulence of Penicillium expansum
Source: Foods. 2025 May 28;14(11):1908. doi: 10.3390/foods14111908 (PMC12154198; doi:10.3390/foods14111908)

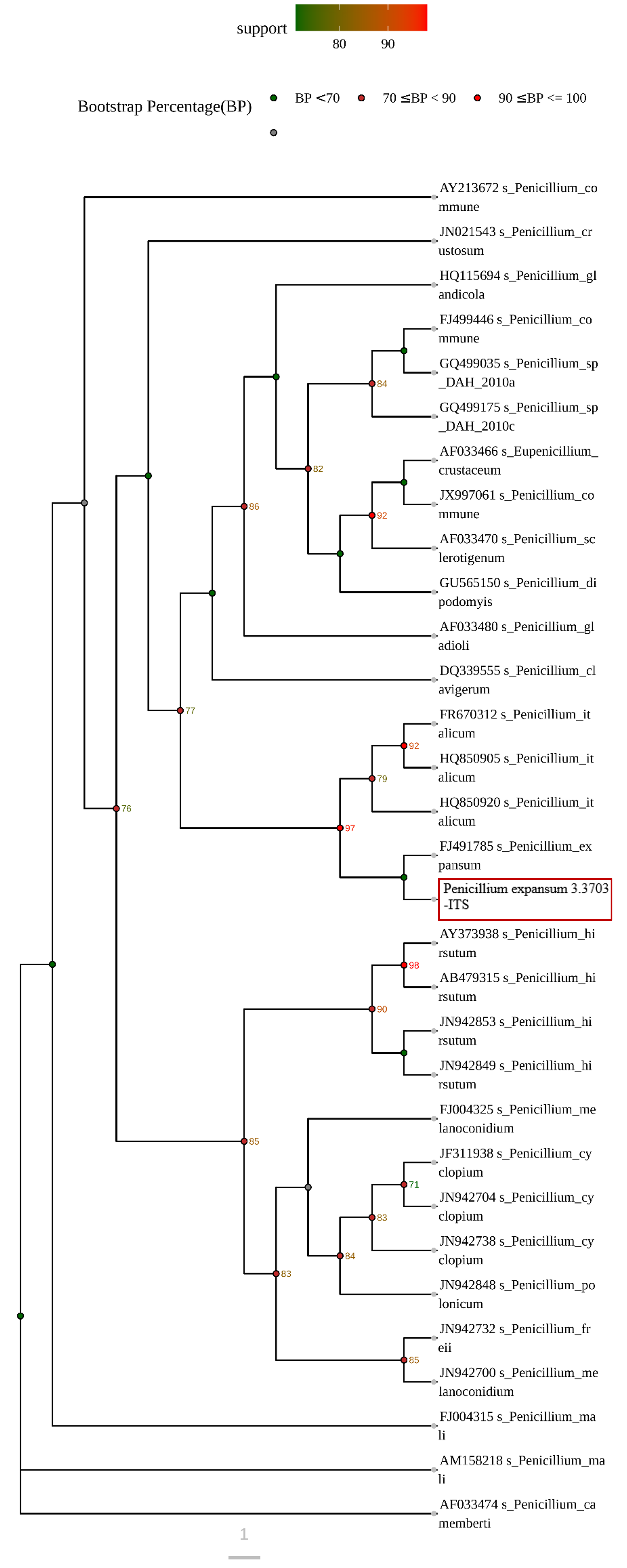

Supplement: Supplementary file 1 [file foods-14-01908-s001.zip › figure S1.tif]

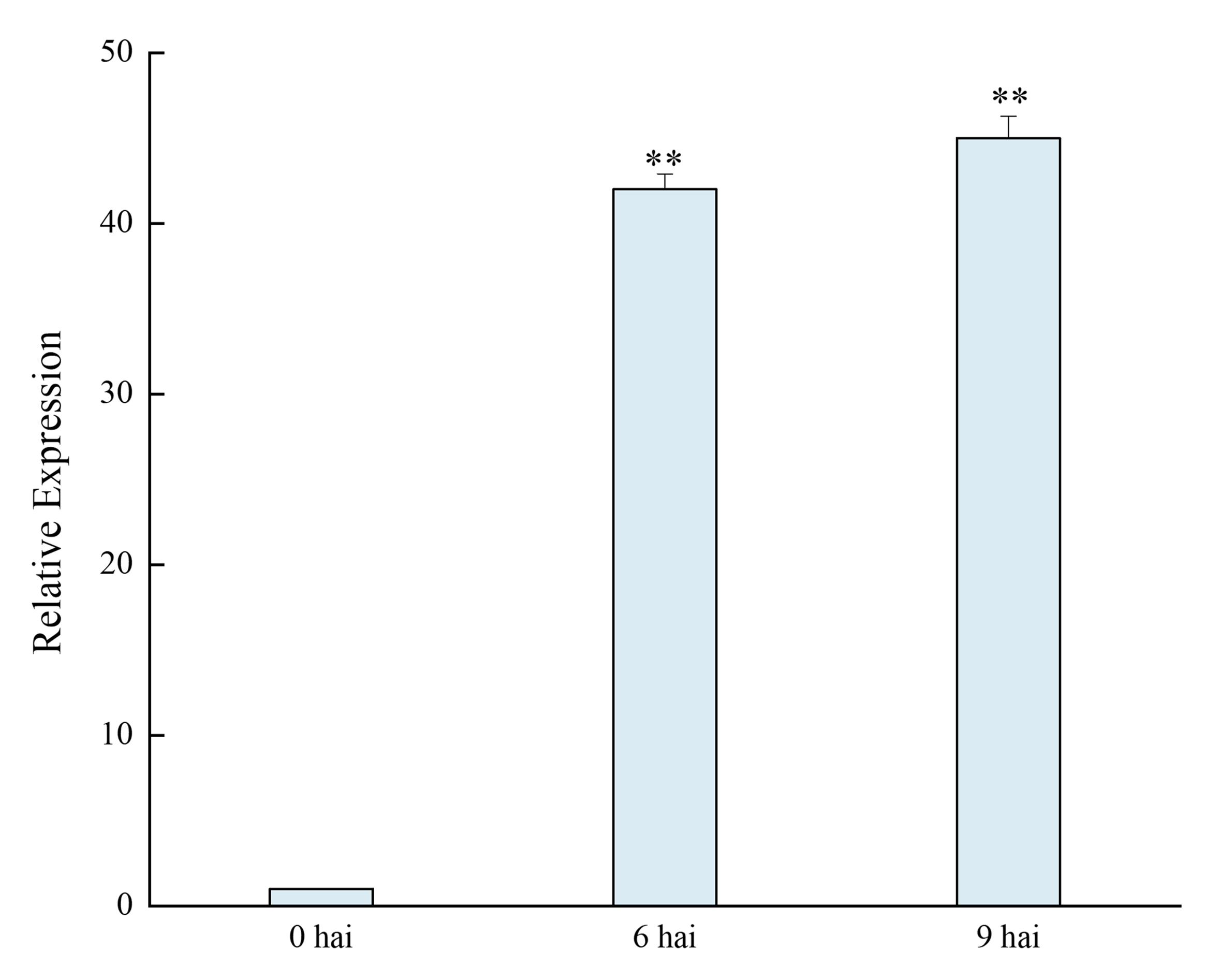

Supplement: Supplementary file 1 [file foods-14-01908-s001.zip › figure S2.tif]

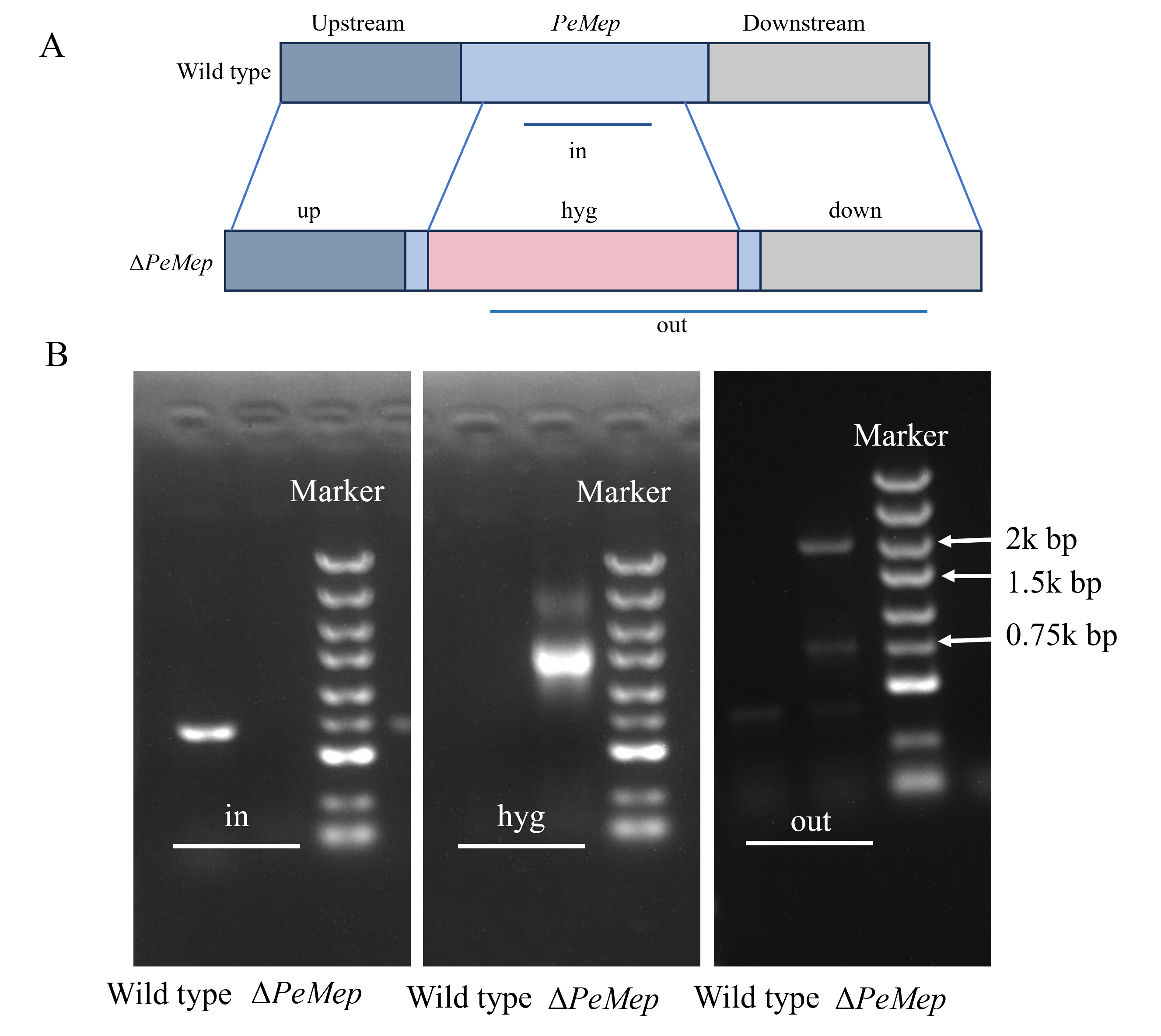

Supplement: Supplementary file 1 [file foods-14-01908-s001.zip › figure S3.tif]
